# Disease-modifying antirheumatic drugs in childhood-onset IgA vasculitis: a large multicenter Latin American study

**DOI:** 10.1016/j.clinsp.2026.101223

**Published:** 2026-10-28

**Authors:** Clara R. Doria, Ricardo N. Machado, Erika Moscarelli, Galo O.P. Rodríguez, Luisa F.C. Forero, Izabel M. Buscatti, Gleice Clemente, Maria T. Terreri, Mayra Dantas, Luciana M. Carvalho, Francisco H.R. Gomes, Natalia Maronese, Adriana R. Fonseca, Marta F. Rodrigues, Lucia M.A. Campos, Adriana M. Elias, Verena A. Balbi, Nadia E. Aikawa, Katia T. Kozu, Sylvia C.L. Farhat, Clovis A. Silva

**Affiliations:** a Pediatric Rheumatology Unit, Instituto da Criança e do Adolescente, Hospital das Clínicas da Faculdade de Medicina da, Universidade de São Paulo (HCFMUSP), São Paulo, SP, Brazil; b Pediatric Rheumatology Unit, Universidade Federal de São Paulo (UNIFESP), São Paulo, SP, Brazil; c Pediatric Rheumatology Unit, Faculdade de Medicina de Ribeirão Preto (FMRP), Universidade de São Paulo (USP), Ribeirão Preto, SP, Brazil; d Rheumatology Division, Instituto de Puericultura e Pediatria Martagão Gesteira (IPPMG), Universidade Federal do Rio de Janeiro (UFRJ), Rio de Janeiro, RJ, Brazil

Immunoglobulin A Vasculitis (IgAV) is the most prevalent systemic small-vessel vasculitis in childhood, characterized by leukocytoclastic vasculitis with predominant deposition of IgA immune complexes, with cutaneous, articular, gastrointestinal and renal involvements. It may also affect the central nervous system, lungs and testicles. Disease-Modifying Antirheumatic Drugs (DMARDs) are generally indicated for severe, persistent, or relapsing manifestations of IgAV, at symptoms onset or during follow-up. Data regarding DMARD use in childhood-onset IgAV(c-IgAV) remain limited to small cohorts focused on specific agents. To our knowledge, no prior study has evaluated DMARD utilization in a large, multicenter Latin American pediatric population.^1^^,^^2^ Therefore, we aimed to evaluate DMARD use in c-IgAV patients, and identify demographic, cumulative clinical and laboratory data associated with these treatments.

This retrospective multicenter study included IgAV patients, followed at four pediatric rheumatology university centers in Brazil. All patients fulfilled the European Alliance of Associations for Rheumatology, the pediatric Rheumatology International Trials organization and the pediatric Rheumatology European Society (EULAR/PRINTO/PReS) classification criteria for IgAV.^3^ The study was approved by the Institutional Ethics Committee of the primary coordinating center (Protocol n° 70,992,423.8.1001.0068), and by the Ethics Committees of all participating institutions. Prior to the start of data collection initiated, a virtual consensus meeting was held with all participating centers to establish uniform definitions for clinical, laboratory, and therapeutic variables.^4^^,^^5^ A clinical feature was considered persistent if it was present for more than 6-weeks, and recurrent if a new manifestation occurred after a complete resolution.^4^^,^^5^ Cumulative data encompassed clinical, laboratory and treatment variables captured at disease onset (defined as within the first three months following diagnosis) and longitudinally throughout follow-up (at 1-, 5-, 10-, and 15-years, and/or at the final clinical evaluation). Demographic characteristics, as well as cumulative features, were compared between patients who received DMARDs and those who did not until the last follow-up visit. Continuous variables, tested for normality via Shapiro-Wilk, are presented as mean ± SD or median (range), and categorical data as n (%). Group differences (with vs. without DMARD) were compared using Student's *t*-test, Mann-Whitney *U*, or Fisher’s exact test. Univariate p-values were Benjamini-Hochberg adjusted (significance: *q* < 0.05); both p- and *q*-values are reported in Table 1. Multivariable logistic regression identified independent factors for DMARD use, reporting adjusted ORs (95% CI) with significance set at p < 0.05.

**Table 1:** Demographic, cumulative clinical, laboratory and treatment features at the last follow-up visit in 687 patients with childhood-onset IgA vasculitis, according to use of Disease-Modifying Antirheumatic Drugs (DMARD).

Cumulative variables	With cumulative DMARD use (n = 34)	Without cumulative DMARD use (n = 653)	p	*q* (FDR)
Demographic data				
Age at diagnosis, years	7.46 (3.08–14.33)	5.79 (0.80–17.42)	**0.001**	**0.005**
Days between age at onset and age at diagnosis	10.5 (0–92)	7.0 (0.0–731)	0.295	0.479
Female sex	16/34 (47.1)	328/653 (50.2)	0.754	0.852
Body mass index, kg/m^2^	22.2 (14.0–30.8)	18.2 (14.9–44.1)	**0.001**	**0.005**
**Cutaneous/Mucocutaneous involvement**				
Purpura	34/34 (100.0)	643/653 (98.5)	0.617	0.779
Petechiae	18/34 (52.9)	295/653 (45.2)	0.384	0.512
Purpura/Petechiae duration, days	29.5 (5.0–180.0)	14.0 (2.0–240.0)	**0.001**	**0.005**
Persistent purpura/Petechiae	12/33 (36.4)	48/636 (7.5)	**<0.0001**	**0.001**
Recurrent purpura/Petechiae	11/34 (32.4)	138/626 (22.0)	0.204	0.379
**Articular involvement**				
Arthritis/Arthralgia	27/34 (79.4)	537/652 (82.4)	0.647	0.782
Number of episodes of arthritis/Arthralgia	1.0 (1.0–7.0)	1.0 (1.0–4.0)	0.347	0.488
Arthritis/Arthralgia duration, days	10.0 (3.0–90.0)	7.0 (1.0–113.0)	0.058	0.168
Persistent arthritis/Arthralgia	2/27 (7.4)	12/524 (2.3)	0.146	0.316
Recurrent arthritis/Arthralgia	4/27 (14.8)	51/521 (9.8)	0.335	0.488
**Gastrointestinal involvement**				
Abdominal pain	24/34 (70.6)	397/653 (60.8)	0.283	0.475
Abdominal pain duration, days	10.0 (1.0–45.0)	7.0 (1.0–846.0)	0.174	0.362
Moderate to severe abdominal pain	7/20 (35.0)	164/337 (48.7)	0.258	0.449
Persistent abdominal pain	0	12/390 (3.1)	1.000	1.000
Recurrent abdominal pain	8/24 (33.3)	79/392 (20.2)	0.127	0.300
Gastrointestinal bleeding	6/24 (25.0)	80/394 (20.3)	0.604	0.779
Bowel intussusception	1/24 (4.2)	1/394 (0.3)	0.114	0.282
**Renal involvement**	30/34 (88.2)	222/653 (34.0)	**<0.0001**	**0.001**
Systemic arterial hypertension	4/31 (12.9)	43/536 (8.0)	0.313	0.488
Nephrotic syndrome	13/28 (46.4)	4/114 (3.5)	**<0.0001**	**0.001**
Hematuria	23/29 (79.3)	178/220 (80.9)	0.805	0.880
Leukocyturia	11/28 (39.3)	96/217 (44.2)	0.689	0.814
Urinary casts	2/27 (7.4)	10/215 (4.7)	0.629	0.779
Proteinuria	29/30 (96.7)	117/219 (53.4)	**<0.0001**	**0.001**
Acute kidney injury	8/30 (26.7)	43/219 (19.6)	0.346	0.488
Chronic kidney disease	3/29 (10.3)	3/219 (1.4)	**0.023**	0.078
Persistent renal involvement	23/29 (79.3)	87/220 (39.5)	**<0.0001**	**0.001**
Recurrent renal involvement	8/29 (27.6)	61/220 (27.7)	1.000	1.000
**Genital involvement**				
Orchitis	1/19 (5.3)	56/370 (15.1)	0.332	0.488
Number of episodes of orchitis	–	1.0 (1.0–4.0)	–	–
Orchitis duration, days	–	5.0 (1.0–30.0)	–	–
**Neuropsychiatric involvement**	1/34 (2.9)	0/653 (0)	0.050	0.153
**Pulmonary involvement**	0/34 (0)	1/653 (0.2)	1.000	1.000
**Ocular involvement**	1/34 (2.9)	2/653 (0.3)	0.141	0.316
**Cumulative treatments**				
Oral glucocorticoid	31/33 (93.9)	306/652 (46.9)	**0.001**	**0.005**
Intravenous glucocorticoid	7/31 (22.6)	81/644 (12.6)	0.107	0.278
Antiproteinuric agent use	26/34 (76.5)	42/650 (6.5)	**<0.0001**	**0.005**
Antiproteinuric treatment duration, days	1062.0 (71.0–6930.0)	180.0 (45.0–1825.0)	**0.001**	**0.005**

Results are presented as median (minimum‒maximum) or n (%). Significant values (p < 0.05) are shown in bold red color. Comparisons were performed using the Mann-Whitney *U test* for continuous variables and Fisher's exact test for categorical variables. FDR, False Discovery Rate; qFDR, Adjusted p-value.

Conventional synthetic DMARD use was identified in n = 34/687 (5%) patients: azathioprine (Imuran), mycophenolate mofetil (MMF, n = 6), cyclophosphamide (n = 6), cyclosporine (Cyclosporine capsules) and methotrexate (Methotrexate). None of them used biologic DMARDs. Of note, n = 5/34 (15%) patients used ≥ 2 DMARDs. Patients receiving DMARDs were significantly older at diagnosis (7.46 [3.08–14.33] vs. 5.79 [0.80–17.42] years, p = 0.001), and had a higher body mass index (22.2 [14.0–30.8] vs. 18.2 [14.9–44.1] kg/m^2^, p = 0.001). The duration of purpura/petechiae was significantly longer in the DMARD group (29.5 [5.0–180.0] vs. 14.0 [2.0–240.0] days, p = 0.001), as well as the persistent purpura/petechiae (12/33 [36.4%] vs. 48/636 [7.5%], p < 0.0001). Renal manifestations (30/34 [88.2%] vs. 222/653 [34.0%]), proteinuria (29/30 [96.7%] vs. 117/219 [53.4%]), nephrotic syndrome (13/28 [46.4%] vs. 4/114 [3.5%]) and persistent renal involvement (23/29 [79.3%] vs. 87/220 [39.5%]) were significantly more frequent in the DMARD group (p < 0.0001 for all), likewise antiproteinuric agent use (26/34 [76.5%] vs. 42/650 [6.5%], p < 0.0001), with a substantially longer treatment duration (1062.0 [71.0–6930.0] vs. 180.0 [45.0–1825.0] days, p = 0.001) (Table 1). In multivariable logistic regression analysis, antiproteinuric therapy (OR = 25.89; 95% CI 1.47–455.06; p = 0.026) and persistent purpura/petechiae (OR = 20.41; 95% CI 1.12–373.24; p = 0.042) were independently associated with cumulative DMARD use.

This was the first large Latin American multicenter study to characterize the cumulative DMARD use of a childhood-onset IgAV population. Only 5% of our patients required these agents, reinforcing the self-limiting course of the disease in most children and adolescents.^6^ Those who escalated to DMARDs were older at diagnosis, in line with data identifying older age at onset as a predictor of refractory disease and of unfavorable renal outcomes.^6^^,^^7^ Persistent purpura/petechiae was the only extrarenal feature independently associated with DMARD use, consistent with the recognition of cutaneous manifestations lasting beyond six weeks as a marker of protracted disease and a trigger for treatment escalation.^8^ In contrast, articular and gastrointestinal manifestations were not significantly associated with DMARD use in this cohort.^6^

The strongest indication for DMARDs was IgAV nephritis. Antiproteinuric therapy was prescribed to three-quarters of the DMARD group and maintained approximately six times longer, marking a persistent proteinuric nephritis, together with the frequencies of nephrotic syndrome and persistent renal involvement. These data delineate a phenotype of chronic proteinuric kidney disease requiring immunosuppression beyond corticosteroids.^6^ Azathioprine was by far the most frequently prescribed agent in our cohort, whereas MMF and cyclosporine were used in a minority of patients. Current international recommendations list azathioprine, MMF and cyclosporine as second-line options combined with glucocorticoids, and reserve cyclophosphamide for nephrotic syndrome and/or rapidly deteriorating renal function, while acknowledging that the supporting evidence derives from small retrospective series.^9^ Our prescribing pattern is consistent with our experience in which azathioprine combined with glucocorticoids and an angiotensin-converting enzyme inhibitor reduced proteinuria within the first six months,^10^ and probably reflects both regional drug availability and this accumulated local experience.

A key strength of this study lies in the strict application of the validated EULAR/PRINTO/PReS classification criteria for IgAV across a large, multicenter Latin American cohort with standardized longitudinal follow-up. However, limitations must be acknowledged. Its retrospective design introduces the potential for information bias due to missing data, the small number of DMARD-treated patients and the heterogeneity of the agents grouped in this category also limit the statistical power of some analyzes, as reflected in the wide confidence intervals of the multivariable model. And because data were gathered exclusively from tertiary academic centers, the findings may reflect selection bias toward severe cases and lack generalizability to secondary care settings.

In conclusion, DMARD use was uncommon in this cohort and was observed in patients with a higher burden of renal and persistent cutaneous manifestations. Further prospective multicenter studies evaluating targeted therapies, including new biologic DMARDs, will be necessary.

## Declaration of generative AI and AI-assisted technologies in the manuscript preparation process

During the preparation of this work the author(s) used Google Gemini exclusively to improve the English phrasing and clarity of the manuscript. After using this tool/service, the author(s) reviewed and edited the content as needed and take(s) full responsibility for the content of the published article.

## Conflicts of interest

The authors declare no conflicts of interest.

## Data Availability

The datasets generated and/or analyzed during the current study are available from the corresponding author upon reasonable request.
